# Neurological Outcome Beyond the ICU in Patients with Severe Acute Brain Injury: A Knowledge gap for ICU Teams

**DOI:** 10.1177/08850666251375411

**Published:** 2025-09-12

**Authors:** Wolmet E. Haksteen, Ruben J. Bax, Daan C. Velseboer, Janneke Horn

**Affiliations:** Amsterdam UMC, University of Amsterdam, Department of Intensive Care, Amsterdam Neuroscience, Amsterdam, Netherlands

**Keywords:** brain injury, intensive care, neurocritical care, neuro-ICU

## Abstract

**Background:**

In patients with severe acute brain injury (SABI) admitted to the Intensive Care Unit (ICU) outcome is often uncertain. Physicians have to decide on continuation or withdrawal of life-sustaining therapy (WLST) in the first weeks. However, long-term neurological outcomes rarely reach the ICU team, limiting the opportunity to learn from decisions. This study aimed to assess the availability of long-term neurological outcome data and from which sources this could be retrieved.

**Methods:**

This single-center retrospective observational study included all ICU survivors with SABI between January first and December 31^st^ 2022. Patient characteristics and neurological outcome data were extracted from the patient records. Neurological outcome was assessed using the Glasgow Outcome Scale Extended (GOSE) within 6 months after admission.

**Results:**

A total of 65 patients were included, the median Glasgow Coma Scale (GCS) score at admission was 6 [IQR 4-7], the most common admission diagnosis was subarachnoid hemorrhage (34%). The mean ICU stay was 8 days (SD 8) and hospital stay 38 days (SD 29). Outcome data could be retrieved from neurology or neurosurgery outpatient clinic notes in 54% of patients, in 12% from the post-ICU clinic and in 2% from other hospitals, rehabilitation facilities and nursing homes. Mortality within 6 months was 26%. A favorable GOSE score (≥ 5) was found in 18 patients (28%), an unfavorable score in 42 patients (64%), and in 5 patients (8%) no outcome could be determined.

**Conclusions:**

Functional recovery outcomes could be extracted from the patient records in nearly all ICU survivors with SABI. However, these outcomes were mostly identified from outpatient clinic notes from the neurology and neurosurgery department and it is uncertain whether these outcomes reach ICU physicians. A clear feedback loop is needed, to ensure ICU teams can learn from long-term patient trajectories and improve future patient care.

## Introduction

Decisions on continuation or withdrawal of life-sustaining treatment (WLST) in patients admitted to the intensive care unit (ICU) with severe acute brain injury (SABI) are among the most complex challenges faced by ICU physicians.^1–4^ A major impediment is the lack of established guidelines for prediction of outcome in these patients, comparable to the structured protocols available for patients with disorders of consciousness following cardiac arrest.^5^ As a result, clinicians rely on their personal expertise and standard diagnostic tools such as CT-scans and bedside neurological examination to estimate the potential for recovery. Furthermore, as patients with SABI cannot communicate their preferences regarding an acceptable outcome, family members are often tasked with expressing the patient's wishes. This puts a heavy burden on the family and the patient's wishes may not always be known or accurately conveyed.^6^ Given these challenges, ICU teams must make decisions with significant consequences in the early period after injury, often with limited certainty on the long-term recovery potential. A contributing factor to this uncertainty is the lack of structured feedback from rehabilitation facilities and nursing homes regarding the long-term outcomes of SABI patients. Once patients are discharged to the ward or transferred to another care facility, opportunities for the ICU team for long-term follow-up become scarce. This is particularly concerning as neurological recovery in SABI patients can be expected in months rather than weeks. The TRACK-TBI study clearly showed that recovery from severe traumatic brain injury (TBI) requires time.^7^ Only 12% of the patients had a favorable outcome 2 weeks post injury, whereas more than half achieved favorable outcome at 12 months. Similarly, Wilkins et al reported that more than 50% of the patients with an unfavorable outcome at 3 months follow-up, recovered to a favorable outcome at 2 years post-injury.^8^ These results highlight the importance of feedback on long-term outcomes to the ICU team members who in the acute phase after SABI have to take decisions. Without this information, important knowledge is lost and physicians lose the opportunity to refine the decision-making process for future patients.

In this retrospective study in ICU survivors with SABI, we aimed to assess the availability of long-term neurological outcome data for ICU physicians. Specifically, we aimed to determine from which sources this information could best be retrieved. Furthermore, we documented which diagnostic tests were conducted to optimize prognostication.

## Methods

### Design

This was a single-center, retrospective, observational cohort study performed at the ICU of the Amsterdam University Medical Centers, location AMC, between January 1, 2022 and December 31, 2022. The study is part of the IMPROVE-DOC protocol approved by the local Medical Ethical Committee (NL82013.018.22).

### Study Population

All adult SABI patients who survived ICU admission. SABI was defined as: traumatic brain injury (TBI), ischemic stroke, subarachnoid hemorrhage, intracranial hemorrhage or meningoencephalitis. Patients with a Glasgow Coma Scale (GCS) score ≤ 8 prior to ICU admission were included. Patients for whom the treating physicians decided to WLST within 24h after ICU admission were excluded. Patients known to have a neurodegenerative disease (i.e. Alzheimer's disease or amyotrophic lateral sclerosis) were excluded as well. A departmental log of all patients admitted to the ICU initially assigned to the departments of neurology, neurosurgery or traumasurgery was used to identify eligible patients.

### Patient Characteristics

All patient characteristics were extracted from the electronic patient records and encompassed demographic data i.e. age, sex, and medical history, along with medical data i.e. admission diagnosis, GCS score at admission, pupillary reactivity, highest SOFA score, readmission to the ICU, length of hospital and ICU stay, mortality, CT-scans, EEG recordings and MRI-scans. The EEG recordings were all standard registrations with a duration of approximately 30–60 min. Patient performance prior to hospital admission was assessed using the clinical frailty score (CFS). The CFS was divided into two categories: “Frail” (CFS score ≥ 5) and “Non-frail” (CFS score ≤ 4).^9^ The CT-scans were evaluated by an experienced neuro-radiologist and/or a neuro-intensivist for highly pathological findings as previously defined by Amiri et al.^10^ These highly pathological findings encompassed a Fischer score ≥ 3 (for subarachnoid hemorrhage), a Marshall classification ≥ 3 (for TBI), intracerebral hemorrhage volume of ≥ 30 milliliter, strategic hemorrhage or infarct in the brainstem (for ischemic stroke or infratentorial hemorrhage), or global cortical oedema. Neurological outcome was assessed using the Glasgow Outcome Scale Extended (GOSE), with the highest score within 6 months inferred from data extracted from the medical records. If a functional or quality of life score other than the GOSE score was available, such as the EuroQol-5D or Modified Rankin Scale, it was translated to a corresponding GOSE score. In case of uncertainty, a second reviewer (an experienced neuro-intensivist) was involved to ensure accuracy in determining the outcome.^11^ A GOSE score of ≥ 5 was considered a favorable outcome. Additionally, the type (in-person, telephone encounter or videoconference) and the number of post-discharge visits was documented.

### Statistical Analysis

Continuous data with nonparametric distribution are reported as medians and interquartile ranges [IQR], while continuous data with parametric distribution are presented as means and standard deviations (SD). Categorical variables are reported using frequencies and percentages. All analyses were performed using R (version 4.2.3).

## Results

**Figure 1.**
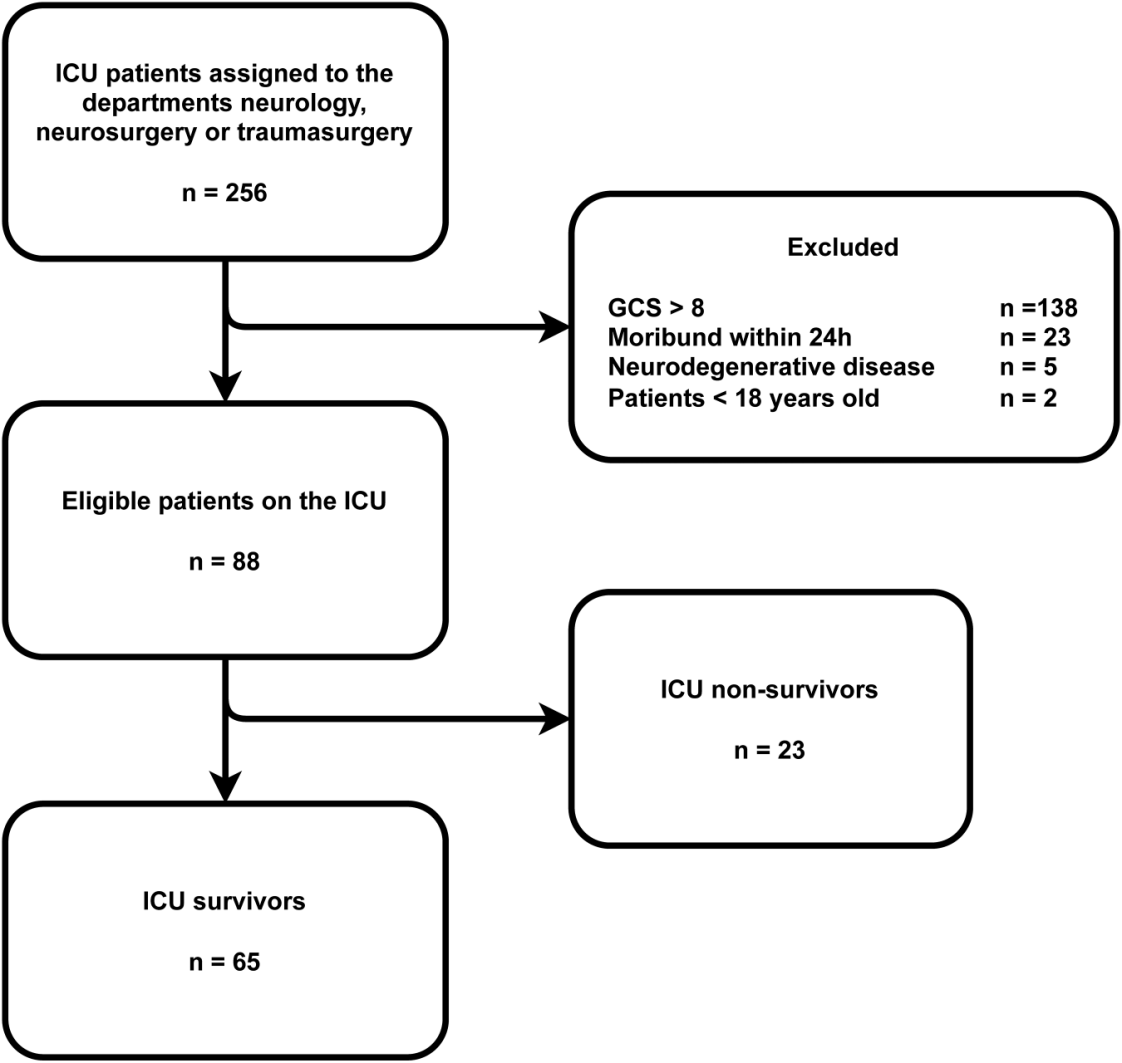
Flowchart depicting all patients screened for eligibility between January 1, 2022 and December 31, 2022. GCS = Glasgow Coma Scale score, ICU = Intensive Care Unit.

**Figure 2.**
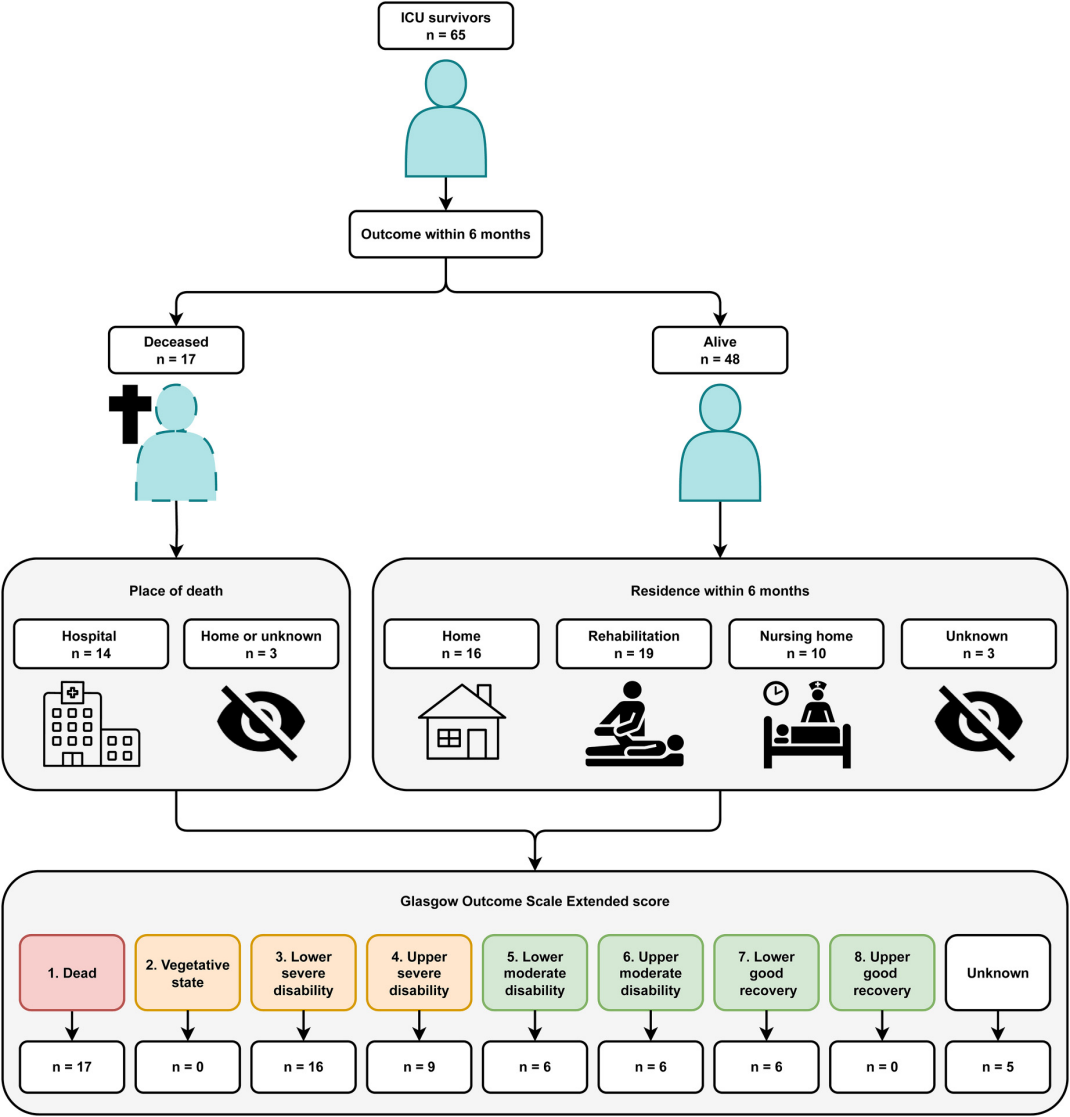
Neurological outcome and residence of ICU survivors with severe acute brain injury identified from the electronic patient records within 6 months after hospital admission. ICU = Intensive Care Unit.

A total of 256 patients with acute brain injury were reviewed for eligibility, 65 patients were included in this study (Figure 1). Baseline characteristics are depicted in Table 1. The GCS score at admission was low, median 6 [IQR 4-7]. The admission diagnosis most frequently found was subarachnoid hemorrhage (34%), followed by TBI (25%) and intracranial hemorrhage (25%). The highest SOFA score during ICU admission was a median score of 10 [IQR 8-11]. In 61 patients (93.8%) one or more neurosurgical interventions were performed. The most common was placement of an external ventricular drainage catheter (70.8%), followed by decompressive hemicraniectomy (29.3%) and coiling (27.6%). The frequency and timing of diagnostic assessments used during hospital admission can be found in Table 2. In all patients admitted to the ICU with SABI a CT-scan was performed at the ER, highly pathological findings were present in 89%. An MRI scan of the brain was performed in 10 patients. The mean ICU stay was 8 days (SD 8) and the mean hospital stay was 38 days (SD 29). A total of 34 patients were discharged from the hospital to a rehabilitation facility, 6 to a nursing home and 8 patients were discharged to their homes. Of 3 patients the discharge location could not be retrieved. Figure 2. depicts both the residence and neurological outcome identified within 6 months after hospital admission. A favorable GOSE score was found in 18 patients (28%), and an unfavorable in 42 patients (64%). In 5 patients (8%) no GOSE score could be determined from the information found in the patient records. Mortality within 6 months after hospital admission was 26%, 3 patients died after WLST, the remaining patients died from clinical deterioration. The median timing of outcome identification was 126 days after hospital admission [IQR 49-169]. In 54% of patients the GOSE score was determined from notes of the neurology or neurosurgery outpatient clinic and in 12% from the post-ICU clinic. Among these patients, the notes were based on post-discharge telephone encounters in 43 patients and on an in-person consultation in 9 patients. For 2% of the patients the outcome was obtained from documentation from other hospitals, rehabilitation facilities and nursing homes and in 8% no outcomes could be identified. A GOSE score of 1, the non-survivors (26%) was determined from the documentation of the ward, outpatient clinic notes, and automatically generated notifications of death in the electronic patient record if the patient died after hospital discharge. The median number of in-person post-discharge visits was 1 [IQR 0-2], the median number of telephone encounters was 3 [IQR 2-4] and the use of videoconference did not occur.

**Table 1.** Baseline Characteristics.

	n = 65
Age (years)	60 [42-67]
Sex (female)	28 (43.1%)
Glasgow Coma Scale at admission	6 [4-7]
Reactive pupils at admission	
Both	48 (73.8%)
One	8 (12.3%)
None	4 (6.2%)
Unknown	5 (7.7%)
Admission diagnosis	
Subarachnoid hemorrhage	22 (33.8%)
Traumatic brain injury	16 (24.6%)
Intracranial hemorrhage	16 (24.6%)
Ischemic stroke	10 (15.4%)
Meningo-encephalitis	1 (1.5%)
Severity scores	
Hunt and Hess score for subarachnoid hemorrhage	4 [4-5]
Intracerebral hemorrhage score	3 [2-3]
NIHSS score for stroke	29 [28-30]
Clinical Frailty Scale score	
Frail	4 (6.1%)
Nonfrail	51 (78.5%)
Unknown	10 (15.4%)

*Values are presented as count (percentages) or median [interquartile range]. “Frail” is defined as a CFS score* ≥ *5, “Nonfrail” is defined as a CFS score* ≤ *4. ICU* *=* *Intensive Care Unit, NIHSS score* *=* *National Institutes of Health Stroke Scale score.*

**Table 2.** Diagnostic Assessments Conducted During Hospital Admission.

	n = 65	
CT-scans		(New) highly pathological findings present (n, %)
At the ER	65 (100%)	58 (89.2%)
Scan during ICU admission	42 (64.6%)	20 (47.6%)
Scan after interruption of sedation	23 (35.4%)	6 (26.1%)
Timing CT-scans (days)		
Scan on the ICU	1 [0-2]	
Scan after interruption sedation	6 [3-9]	
MRI	10 (15.4%)	
Timing MRI (days)	18 [3-23]	
EEG		
One	10 (15.4%)	
Two	6 (9.2%)	
Timing EEG (days)		
First	9 [7-13]	
Second	18 [13-26]	

*Values are presented as count (percentages) or median [interquartile range]. Highly pathological findings were defined as: Fischer score* ≥ *3 (for subarachnoid hemorrhage), Marshall classification* ≥ *3 (for TBI), intracerebral hemorrhage volume* ≥ *30 milliliter, strategic hemorrhage or infarct in brainstem (for ischemic stroke or infratentorial hemorrhage), or global cortical oedema. CT-scan* *=* *Computed Tomography scan, EEG* *=* *electroencephalography, ER* *=* *Emergency Room, ICU* *=* *Intensive Care Unit, MRI* *=* *Magnetic Resonance Imaging.*

## Discussion

This study demonstrates that while most patients were discharged to rehabilitation facilities, information on outcomes was primarily available from outpatient clinic notes from the neurology and neurosurgery departments. Only a small proportion of patients was contacted via the post-ICU clinic, 2 to 3 months after hospital discharge. Documentation from rehabilitation facilities and nursing homes was largely absent, limiting comprehensive insight into long-term patient outcomes. As a result, the ICU team is unlikely to receive any feedback on long-term patient outcomes.

Based on our results and recent literature, we would recommend a three-step approach to improve neurocritical care for SABI patients. First, optimize prognostication, especially for patients with prolonged disorders of consciousness, by using multimodal assessments consisting of bedside neurological examinations, advanced imaging techniques (e.g. functional MRI), EEG with reactivity testing and blood biomarkers.^12,13^ Second, organize a post-ICU clinic, specifically designed for neurocritical care patients.^14,15^ Third, actively inform clinical teams in the ICU about long-term outcomes to better understand recovery trajectories in these patients.

To address the first part of our recommendation, our results show that the use of additional diagnostic tests for optimal prognostication was limited. In only a few patients an MRI was performed, typically more than two weeks after hospital admission. Recent literature does indicate a role for MRI in neurological outcome prediction in patients with SABI.^16–19^ These studies report variability in the timing of imaging, with a median of approximately 9 days after brain injury in some cases, while others suggest performing an MRI later in the trajectory, around two weeks to a month post-injury. Similarly, EEGs were performed in a small number of patients despite increasing evidence for its value in prognostication.^20–22^ Another important development in the field of prognostication is the use of blood biomarkers. Biomarkers such as neurofilament light (NFL) and glial fibrillary acidic protein (GFAP) were found to be associated with long-term functional outcomes in SABI patients.^23–25^ These biomarkers could potentially be integrated into a multimodal prognostication protocol.

The second part of our recommendation concerns the optimization of post-ICU clinics. Post-ICU clinics were invented to detect mental- and physical health problems encountered by patients after an ICU admission.^26–28^ Assessing post-intensive care syndrome in patients with SABI and disorders of consciousness may be difficult, but these patients should not be excluded. Including them in post-ICU clinics may not only benefit patients and families, but also the ICU care team. These clinics possibly provide a feedback loop on clinical outcomes and may create increased awareness in ICU nurses and physicians of patient experiences during ICU admission.^14,29^ If patients themselves are unable to communicate their experiences, families can offer valuable insights. Important questions to ask these families and patients would be: “Are you content with the current state of neurological recovery?” and “Would you have made different choices given the current situation?”. The addition of surveys about quality of life and depression symptoms could aid in answering these questions.^30^ In stroke patients who underwent decompressive hemicraniectomy, quality of life was reduced by 45% overall. However, the majority of patients and their families remained satisfied with the outcome and would have consented to the procedure again.^31,32^ The development of a post-ICU clinic specifically for neurocritical care patients also offers the opportunity to implement outcome assessments tailored to SABI, such as the GOSE score for TBI patients and the Modified Rankin Scale for stroke patients. In addition, establishing a combined outpatient clinic in collaboration with the neurology or neurosurgery departments could further improve patient care. During hospitalization, the care for these patients is multidisciplinary, involving close cooperation between the ICU, neurology, neurosurgery and rehabilitation teams. Extending this integrated approach into the post-discharge phase could ensure continuity of care, improve long-term outcomes and provide a more comprehensive follow-up suited to the needs of neurocritical care patients.^15^ Since functional recovery in SABI patients requires time, post-ICU follow-up programs should consider a revised timeline, ranging from 6 to 12 months.

Lastly, it is important to actively inform ICU teams about the neurological outcomes of their patients. Most patients in our study died due to acute clinical deterioration. This is in contrast with the patients who died during the ICU stay, as nearly all of those patients died following WLST. In a substudy of the TRACK-TBI, the potential for survival and neurological recovery in patients who died after WLST was investigated.^33^ Propensity score matching was used to pair participants with WLST to patients with a similar probability of WLST, based on demographic and clinical characteristics, but for whom treatment was continued. The results suggested a substantial proportion of patients with TBI and WLST may have survived and achieved partial independence (GOSE ≥ 4) at 12 months. This suggests that patients in whom life-sustaining therapy was withdrawn on the ICU may still have had a potential for recovery. Clinicians may underestimate the chance of recovery in SABI patients, as has been previously demonstrated in studies on severe TBI patients.^7,34^ The implementation of a post-ICU clinic for neurocritical care patients could provide valuable insights into functional outcomes, helping to inform ICU teams about the long-term recovery trajectories of their patients. To further support this feedback loop and promote multidisciplinary collaboration, we propose organizing quarterly or semiannual meetings with all healthcare professionals involved in the care for SABI patients to review and discuss the clinic's findings.

### Strengths and Limitations

This study took place in a large neurosurgical center and highlights an issue which may also be present in other medical centers. A limitation of this study is the retrospective design, the GOSE scores were inferred as these scores are not routinely used and documented in current standard care by the post-ICU clinics, neurology- and neurosurgery department in our hospital. The neurological outcome data would be of better quality if scored at set time points and in standardized manner. Additionally, documentation from rehabilitation facilities and nursing homes was found to be largely unavailable, limiting comprehensive insight into long-term neurological outcome.

## Conclusion and Recommendations

Functional recovery outcomes could be extracted from the patient records in nearly all ICU survivors with SABI. However, it is uncertain whether these outcomes are systematically communicated to ICU physicians. Establishing multidisciplinary post-discharge clinics could further enhance the care for neurocritical care patients and provide clinical teams with valuable feedback, enabling them to learn from past decisions and improve future practice.
